# Development of a Novel, Affordable, and Accessible Simulation for Teaching Rotator Cuff Repair

**DOI:** 10.7759/cureus.91069

**Published:** 2025-08-26

**Authors:** Lainey G Bukowiec, Aaron Damon, Julia E Todderud, Mark E Morrey

**Affiliations:** 1 Department of Orthopedic Surgery, Mayo Clinic, Rochester, USA; 2 Multidisciplinary Simulation Center, Mayo Clinic, Rochester, USA; 3 Department of Medicine, Washington State University, Spokane, USA

**Keywords:** arthroscopic surgery, orthopedic trainer, residency training, rotator cuff repair, surgery simulation, surgical education

## Abstract

This study describes the development of a novel, affordable, and accessible simulation model designed to teach arthroscopic rotator cuff repair to orthopedic surgery trainees. The model was created using commercially available, cost-effective products to provide a portable representation of an in vivo intraoperative rotator cuff repair. The technique was developed at a major academic tertiary care center. The rotator cuff repair simulator was designed with clear learning objectives to help train orthopedic residents in shoulder arthroscopic skills, including proper anchor placement, bimanual instrument handling, and knot tying. Key components of the design include a tactile surface for anchor placement, tendon attachment with adjustable tension, and a simulated intraoperative environment using standard arthroscopic portals. The simulator was constructed using household items at a low cost. Trainees can access instructional videos to guide them in constructing the simulator and performing rotator cuff repairs. This innovative model provides a cost-effective, “at-home” simulator for honing essential surgical skills related to arthroscopic rotator cuff repair. Given the challenges of intraoperative training and the impracticality of expensive lab-based models, this surgical training technique offers a financially viable and effective alternative. By providing residents and fellows with an affordable yet high-quality training solution, the simulator supports equitable access to comprehensive surgical rehearsal exercises. It allows hands-on practice in rotator cuff repair within a low-risk, controlled environment. Consistent simulation and practice improve surgical proficiency and confidence, ultimately enhancing patient outcomes by refining the technical skills of future surgeons.

## Introduction

Surgical education has advanced notably with the integration of surgical simulation into the training of aspiring surgeons [1,2]. Simulation-based training (SBT), including simple nonanatomic models, advanced anatomic models, robotic or augmented reality simulators, and cadaveric models, has shown promising results in improving surgical skills and patient outcomes [3,4]. Previous research has demonstrated reduced errors in a variety of surgical procedures following simulation training [5]. Arthroscopic simulator training, in particular, has been shown to enhance surgeon performance, with randomized clinical trials reporting improvements in patient outcome measures closely linked to technical skill [6].

The education of residents and fellows, especially in surgical specialties, is of great importance but is often limited by restricted access to educators and educational resources. Additionally, case volume and the level of trainee participation vary significantly across programs. The traditional master-apprentice paradigm faces challenges in arthroscopic surgery training, as individual learners often present with distinct deficiencies in foundational arthroscopic skills. Remediation may require time-intensive practice outside the operating room to optimize learning. Importantly, operating during the initial skill acquisition phase has been associated with increased operative errors, underscoring the need for strong foundational training opportunities outside the operative setting [7-10].

SBT offers the advantage of allowing surgeons-in-training ample time and resources to acquire knowledge, skills, and behaviors in a controlled environment [10]. These elements are pivotal to success in the operating room. The primary objective of SBT is to enhance patient safety by ensuring competency before trainees perform procedures on real patients.

However, virtual/augmented-reality and laboratory-based anatomic trainers are often impractical for large-scale or repeated practice due to limited access and high costs, both of which present obstacles to training [11]. In contrast, cost-effective, dry, low-fidelity trainers have been shown to improve novice performance in completing basic arthroscopic procedures [12]. Dry simulators have also been demonstrated to reduce task completion time and improve the quality of performance among surgical trainees, supporting their broader development and use [12].

In light of these challenges and opportunities, this study aims to develop a dry shoulder training model designed to teach orthopedic surgery trainees how to perform arthroscopic rotator cuff repair. The simulator addresses accessibility, flexibility, and financial barriers commonly faced by orthopedic residents and fellows. It provides a portable, easily repeatable modality for skill acquisition and improvement over time, using household instruments to increase access and dramatically reduce costs. To our knowledge, no other simulators have been documented that combine affordability with accessible, skill-based training. The interactive features of this simulator are designed to engage learners by providing a dynamic, moderate-fidelity platform tailored to the diverse needs of surgical trainees and training programs.

## Technical report

Prior to the construction of the shoulder simulator, the primary learning objectives of its design were clearly established. Defining such objectives has been shown to improve the educational value of simulators by directing learning toward specific task training and intentionally developing models with skill acquisition as the primary focus [13,14].

The primary learning goals of the simulator were to train junior orthopedic residents in the foundational skills required to perform a rotator cuff repair, including proper anatomic placement and angulation of anchors, bimanual instrument handling, portal switching, suture management, suture passage, arthroscopic shuttling, and arthroscopic knot tying.

To address these objectives, key design components included a representative tactile surface anatomy of the humeral head for proper anchor placement, replication of the cortico-cancellous interface for anchor deployment, the presence of a tendon with adjustable tension to simulate different retraction parameters, and replication of an intraoperative environment using standard arthroscopic portals.

Simulator construction

**Figure 1:**
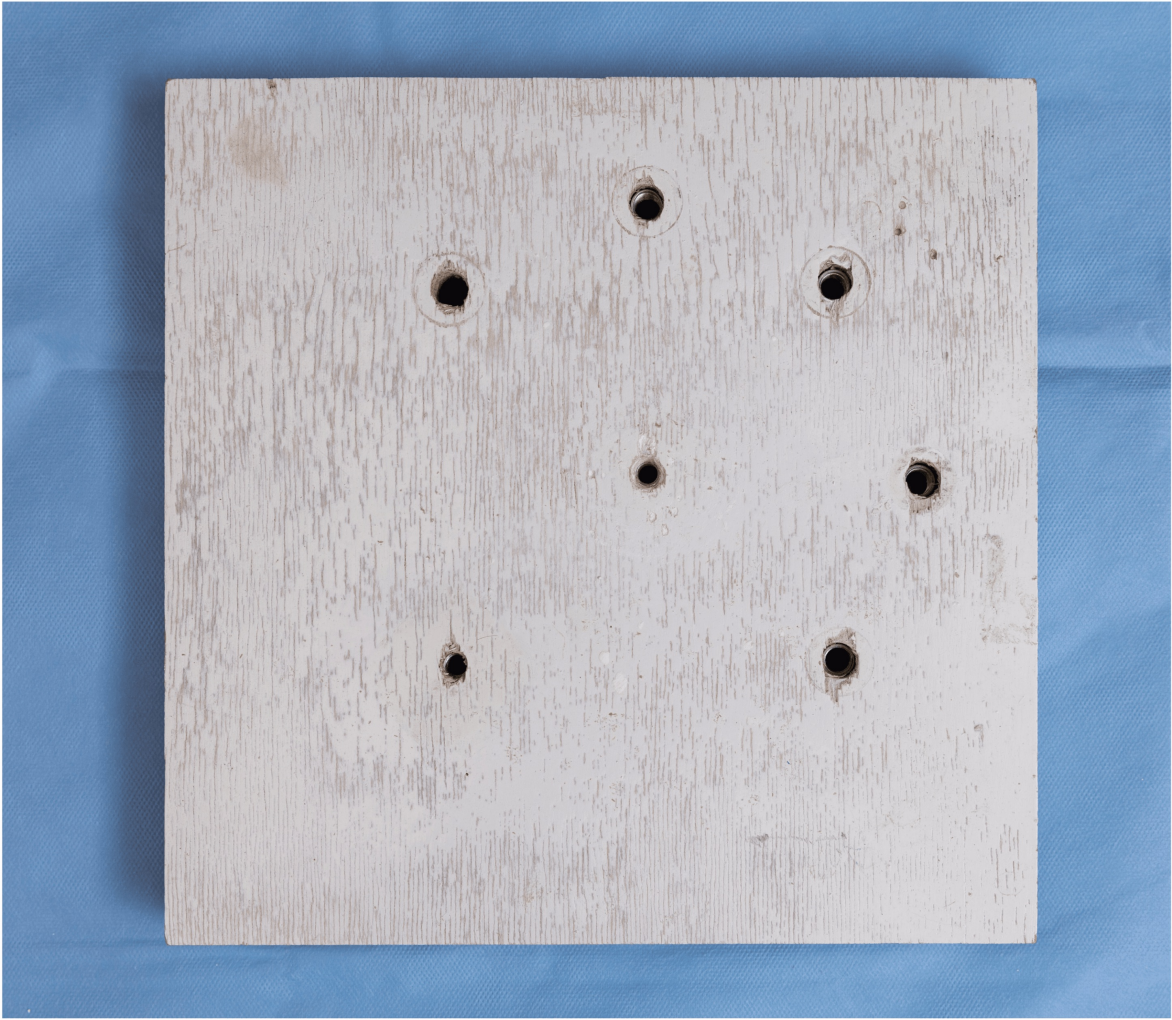
Foundational base with drilled holes

The foundational base of the simulator was constructed from ½-inch plywood, cut with a standard table saw fitted with a wood-cutting blade (Figure 1). A 9 × 9-inch piece was cut to form the base of the trainer. A hand drill with a 3/8-inch standard wood drill bit was used to create five arthroscopic portal holes, while a 1/4-inch drill bit was used to create the remaining two holes.

**Figure 2:**
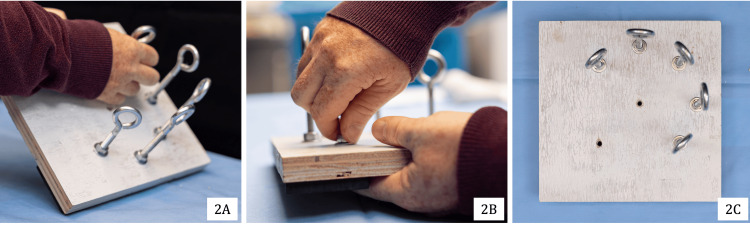
Construction and placement of simulated arthroscopic portals: portal arrangement (2A), placement of the portals (2B), and portal distribution and alignment (2C)

**Figure 3:**
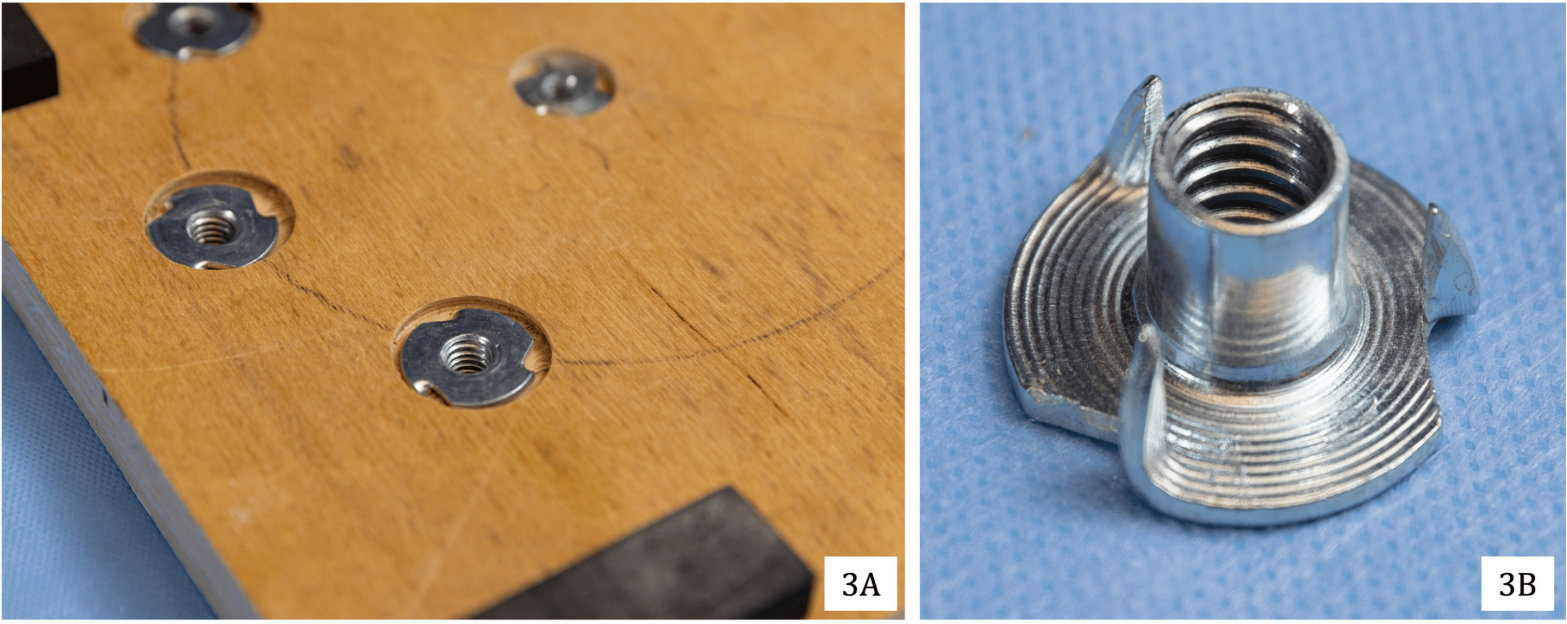
T-nuts used to secure instrumentation to the back of the foundational base: placement of the T-nuts in the base (3A) and T-nuts utilized for the model (3B)

To replicate the restrictions present in arthroscopic surgery, simulated arthroscopic working portals were created by affixing five National ¼ × 3-inch stainless steel eye bolts (#N221-580) to the top of the foundational base. They were positioned along the circumference of a circle with a radius of 3 inches, centered on a central hole (Figure 2), simulating the average global working area for shoulder arthroscopy. The five eye bolts were spaced roughly 2 inches apart along the circumference. All instrumentation in rotator cuff surgery was inserted through the eye bolts to replicate arthroscopic instrument navigation through portals. The eye bolts were secured to the foundational base using ¼″-20 stainless steel four-prong T-nuts (Figure 3).

**Figure 4:**
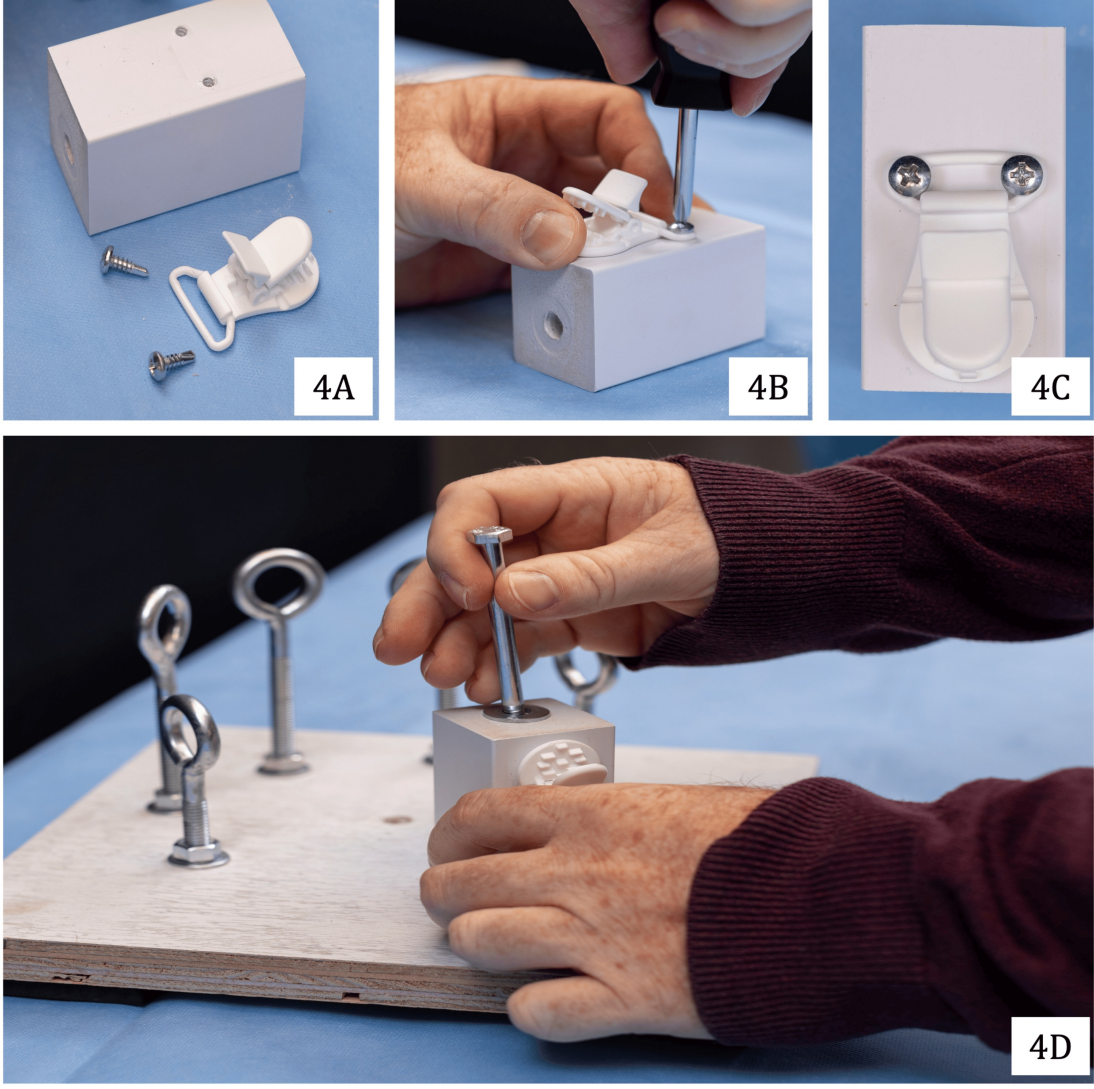
Assembly of wooden post to suspend simulated rotator cuff tissue: post components (4A), assembly (4B), completed view (4C), and fixation to the base (4D)

To stabilize the simulated rotator cuff during repair, a small wooden post (2¾ × 1½ × 1½ inches) was affixed to a plastic pacifier clamp using standard ¾-inch wood screws. The constructed wooden post was then secured to the remaining hole within a 3-inch radius adjacent to the central hole on the foundational base. One ¼-20 × 4-inch bolt was used to anchor the post to the base with a ¼″-20 stainless steel four-prong T-nut. Construction of the post is shown in Figure 4.

**Figure 5:**
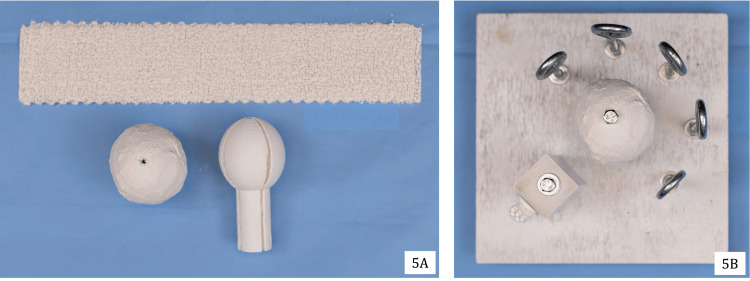
Assembly of humeral head with plaster (5A) and placement on the foundational base (5B)

The humeral head was crafted from Craft Wrap Plaster Cloth (Plaster of Paris), wrapped sequentially around a mold of a humeral head in multiple layers (Figure 5). For an inexpensive option, the mold can be constructed from household items, such as a PVC pipe and end cap, to replicate a circular head with a cylindrical base (Figure 5, left). Alternatively, the humeral head may be 3D printed. As 3D printing becomes more cost-effective, this option may be increasingly feasible and widely available, depending on the resources of the trainee or program.

The prototype in this study utilized an Ultimaker 5S 3D printer (Ultimaker B.V., Utrecht, Netherlands). Polypropylene filament (2.85 mm diameter) was supplied in a 750-gram roll. An STL (stereolithography) file of a right humeral head was created using open-access software Autodesk Tinkercad (v.3d design; 2019). The STL file was processed with Cura software (v.4.0; 2018, Ultimaker) using appropriate printer settings for production.

One Hillman ¼ × 4-inch stainless steel full-thread hex machine bolt was affixed to the center hole of the base. This served as a stable attachment point for the simulated humeral head and was secured with a ¼″-20 stainless steel four-prong T-nut on the opposing side. The plaster humeral head was affixed to the central post by drilling a ⅜-inch hole in its center and attaching it to the upright post using the center bolt (Figure 5).

**Figure 6:**
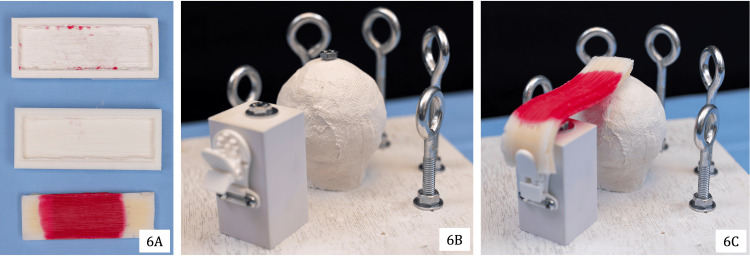
Construction and suspension of simulated rotator cuff tissue: tissue modeling and materials (6A), orientation for placement on the model (6B), and assembled model with tissue (6C)

When clamped, silicone tissue could be draped over the humeral head for access through the simulated portals (Figure 6, right). The simulated tendon was cast in a 3D-printed mold to replicate the thickness, pliability, and appearance of real tissue (Figure 6, left). The mold was filled with Ecoflex-20 silicone (Smooth-On, Inc., Macungie, PA, USA) and dyed with FUSEFX silicone dye S-301-D to create an opaque red color. After curing for four hours, the silicone was removed and ready for use. As a low-cost alternative, standard door and window silicone available at hardware stores (e.g., GE Silicone 1 Clear All-Purpose Sealant, 10.1 oz) may be substituted.

The entire apparatus can be secured to the working table using any commercially available woodworking clamp, providing a stable platform for surgical training.

An instructional video is available that demonstrates how to construct a rudimentary version of the model. This video also presents alternative, readily available options for simulator construction, such as using drywall instead of plaster or a 3D-printed humeral head and an ACE bandage instead of a 3D-printed tendon (Video 1).

**Video 1:** Making the rotator cuff simulator: instructional walkthrough of steps and materials

Instruments

**Figure 7:**
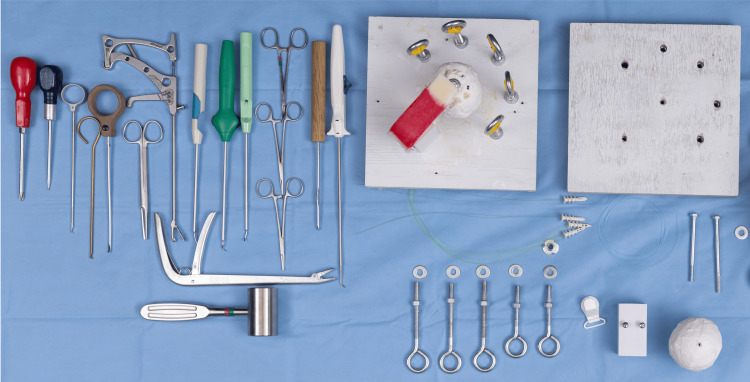
Instruments used during simulation

Instruments can be donated, purchased online, or borrowed from the operating room (Figure 7). Low-cost alternatives have also been developed. For example, simulated cuff graspers and points can be made from fishhook removers, and suture can be substituted with fishing line available at most tackle stores. Awls, screwdrivers, clamps, and anchors can be obtained from standard hardware stores.

The only required instrument that cannot be inexpensively substituted is a suture-passing device. A suture shuttle device is preferred, as it allows practice with manipulation and shuttling techniques. However, alternative “fast pass” devices may also be used to provide familiarity with different approaches for passing a suture through a tendon.

Simulator use

An instructional video is provided demonstrating various foundational skills, proper use of the model, and rotator cuff repair techniques (Video 2).

**Video 2:** Sample of hands-on practice with the model, including simulated skills such as anchor placement, arthroscopic knot tying, and instrument management

Piercing the plaster humeral head with a starting awl and mallet allows the creation of pilot holes for anchor placement during the rotator cuff repair simulation. Anchors are fashioned from standard nylon self-drilling drywall anchors. Two holes are drilled into the end of each anchor with a standard 1/32-inch drill bit to create a sliding hole. High-tensile fishing line (50-pound test) is threaded through these holes to create a preloaded anchor simulating a sliding single- or double-loaded suture anchor. Anchors are placed into the pilot holes on the humeral head using a standard Phillips screwdriver.

Following this setup, trainees can practice the complete sequence of a rotator cuff repair, including instrument handling, suture management, and tissue manipulation through the arthroscopic portals, as well as the creation of shuttle and arthroscopic knots for a variety of repair constructs, including single-row and double-row repairs.

Two steps must be performed outside the portals. The first is punching and placement of the drywall anchors with a Phillips screwdriver, simulating placement through an accessory portal. The second is cutting the suture at the conclusion of the simulation. After each practice session, the pilot holes can be filled with spray foam insulation, allowing the humeral head to be reused for multiple subsequent attempts. The silicone tissue can also be reused multiple times but is ultimately disposable once it loses structural integrity after repeated punctures.

## Discussion

A novel dry rotator cuff model was developed as a cost-effective simulation tool to teach orthopedic surgery trainees arthroscopic rotator cuff repair.

*As a second-year resident, I found immense value in using the rotator cuff repair simulator. It provided hands-on learning that textbooks cannot match. With each attempt, I saw tangible improvements in how I handled the instruments and the time it took me to complete the procedure. The simulator not only accelerated my learning curve but also boosted my confidence in my skills, making it an invaluable tool in my surgical training* - Surgical trainee

*Residents and fellows who use this model increase their facility, automaticity, and efficiency with rotator cuff repairs. It allows trainees to learn different repair constructs and techniques and improves their execution of these techniques in the operating room. The ability to have multiple repetitions over time builds skills and interrupts the forgetting curve *- Supervising surgeon

Comparison to existing simulations for rotator cuff repair

Rotator cuff repair simulation can be approached through a variety of modalities, each with distinct advantages and limitations (Table 1).

**Table 1:** Various arthroscopic simulation modalities and their associated costs

Simulation modality	Typical cost per simulated procedure (including acquisition of materials)
Cadaver lab	$4,000-7,000
Virtual simulator	$260,000
Anatomic simulator	$1,200-3,000
Nonanatomic simulator	$825-1,060

Cadaver labs provide unmatched exposure to real anatomy. However, their utility is restricted by the high costs of cadavers, implants, and instruments, along with legal restrictions and nonreusability [15]. Additional time and expense are required for anatomic dissection and for creating cuff tears, given the variability of cadaveric specimens. Access is therefore limited to programs with sufficient donors and legal authority and is often unavailable in resource-limited settings.

Virtual simulators offer realistic anatomy and the opportunity to practice advanced skills [16]. The main drawbacks include their high cost, limited accessibility depending on institutional resources, and lack of portability. Because these simulators are fixed to specific locations, trainees may have limited opportunities for consistent practice.

Anatomic training models provide semi-realistic simulations of advanced tasks while offering immediate feedback on technique. Their portability allows for greater flexibility in training locations. However, they require spare parts, an appropriate camera system to mimic arthroscopy, and still carry relatively high costs. While generally less expensive than cadavers or virtual simulators, they remain a substantial financial investment (Table 1).

A final simulation modality is nonanatomic simulators, which are valued for their affordability, portability, and suitability for repeated practice outside of the operating room. These models provide an accessible platform for the acquisition of basic skills; however, their limitations include reduced fidelity and the inability to fully replicate the complexities of arthroscopic surgery.

Cost analysis of the current model

Assuming trainees already have access to a basic toolkit (hammer, wrench, and screwdriver), the total cost of constructing this simulator is approximately $53. A detailed breakdown of individual component costs is provided in Table 2.

**Table 2:** Breakdown of cost to create the rotator cuff simulation model

Item	Cost
Project plywood (2’ × 2’)	$6.49
¼-20 × 4” hex bolt	$1.38
¼-20 washers (×6)	$1.25
Plaster of Paris	$8.49
3D molds for plaster application	~$1.50
Pointed-tip chisel	$6.12
C-clamp	$5.74
¼-20 eye bolts (×5)	$2.60
Plastic pacifier clamp	$1.72
Shoulder tissue (silicone)	$8.98
Hammer	$6.97
Phillips-head screwdriver	$1.29
Suture or fishing line	$0.49
Wall anchors (×2)	$0.29

Considerations and lessons learned

The simulator provides excellent haptic feedback and effectively facilitates learning the skills and steps required to complete a rotator cuff repair. The instructional video (Video 2), which demonstrates proper execution of these skills and techniques, offers trainees on-demand access to clear guidance. In addition, the ability to “cover” the model and use it with an inexpensive camera or scope tower adds flexibility depending on available resources. Reusability of the simulator is also excellent.

This study describes the development of a low-cost, accessible rotator cuff repair simulator with the potential to enhance orthopedic surgical training. It outlines the construction of the model, required materials, benefits for surgical trainees, and applications in training. The model addresses the growing need for cost-effective training tools as alternatives to prohibitively expensive methods. Using readily available materials, this reusable simulator closely replicates essential skills while allowing repeated practice and continued training opportunities.

As evidence continues to support the importance of high-quality, consistent surgical training outside the operating room to optimize patient outcomes, this simulator helps bridge the gap by providing an affordable, effective platform for skill development. The ability to perform repeated exercises in anchor placement, arthroscopic knot tying, and tendon manipulation in a controlled, low-risk environment reinforces deliberate practice, which has been shown to significantly improve surgical competency [17]. The primary benefits of this model lie in its accessibility, low cost, and ease of construction, making it especially valuable for training programs with limited resources or case volumes.

Limitations

Although this model provides valuable tactile feedback, it lacks the complexity and fidelity of more advanced training methods, such as cadaveric dissection or virtual reality simulators. While designed to be reusable, components like the silicone simulated tissue degrade over repeated use due to punctures and wear, requiring periodic replacement. Variability in assembly may contribute to differences in quality or function, though these issues can be mitigated through the use of the instructional video.

Another limitation is the absence of real-time feedback mechanisms to evaluate trainee performance. The feedback presented in this study is largely anecdotal and would be strengthened by structured assessments or formal evaluations of surgical competency. Future directions could include the collection of quantitative data on skill acquisition, learning curves, and trainee satisfaction.

## Conclusions

The development of a cost-effective and reusable arthroscopic rotator cuff repair simulator represents an incremental yet meaningful advancement in surgical training for orthopedic residents and fellows. Unlike traditional models, this simulator offers a pragmatic, economically viable alternative that is also reusable. Its ability to replicate intraoperative scenarios provides a valuable platform for trainees to practice and refine their skills using spaced repetition and graduated levels of “desirable difficulties,” which have been shown to improve procedural skill retention.

Importantly, integrating this simulator into residency and fellowship training programs promotes equitable access for all trainees, transcending financial barriers and fostering inclusivity. By democratizing access to high-quality training, this innovative, low-cost, moderate-fidelity simulator has the potential to empower surgical learners and ultimately improve patient outcomes through the cultivation of skilled, confident practitioners.
